# Influence of adenovirus and MVA vaccines on the breadth and hierarchy of T cell responses

**DOI:** 10.1016/j.vaccine.2016.07.050

**Published:** 2016-08-31

**Authors:** Christine S. Rollier, Adrian V.S. Hill, Arturo Reyes-Sandoval

**Affiliations:** a Oxford Vaccine Group, Department of Paediatrics, University of Oxford and the NIHR Oxford Biomedical Research Centre, Oxford, United Kingdom; b The Jenner Institute, University of Oxford, Roosevelt Drive, Oxford OX3 7DQ, United Kingdom

**Keywords:** Vaccines, Viral vectors, Subdominant T-cell epitopes, T-cell hierarchy

## Abstract

Viral-vectored vaccines are in clinical development for several infectious diseases where T-cell responses can mediate protection, and responses to sub-dominant epitopes is needed. Little is known about the influence of MVA or adenoviral vectors on the hierarchy of the dominant and sub-dominant T-cell epitopes. We investigated this aspect in mice using a malaria immunogen. Our results demonstrate that the T-cell hierarchy is influenced by the timing of analysis, rather than by the vector after a single immunization, with hierarchy changing over time. Repeated homologous immunization reduced the breadth of responses, while heterologous prime-boost induced the strongest response to the dominant epitope, albeit with only modest response to the sub-dominant epitopes.

## Highlights

• Viral-vectored vaccines are expected to induce T-cell responses to sub-dominant epitopes.
• Hierarchy of T-cell response is influenced by the timing of analysis after a single immunization.
• Repeated homologous immunization reduces the breadth of T-cell response.
• Heterologous prime-boost induces a modest increase of the subdominant responses.

## 1 Introduction

Leading vaccine strategies aiming at inducing strong cellular immunity in humans use recombinant adenovirus (Ad) and Modified Vaccinia Ankara (MVA) [1], [2]. These vectors have been assessed since the late 90s for their capacity to induce different frequency, phenotype, function and localization of T- and B-cell responses [1], [3], [4], [5]. A consensus emerged that priming with Ad followed by boosting with MVA was optimal to maintain strong and long-lived CD8+ T-cell responses [5], [6], and thus many comparisons have been performed in the context of various heterologous prime – boost regimens [7], [8], [9], with few exceptions comparing them individually [3], [10], [11]. In the latter studies, T-cell responses were measured with tetramers [3], dominant epitopes [10], or peptide pools [11], but none investigated the dominance pattern of T-cell responses induced by each vaccine vector. However, it has been suggested that the immunogenicity of the vector is likely to have a strong influence on the transgenic antigen [12]. The objective of this study was to identify the influence of Ad and MVA vaccine vectors on the hierarchy and breadth of the T-cell epitope responses to the vaccine immunogen, and to specifically address the following four questions: (1) Is the hierarchy of T cell responses influenced by the vector? (2) Does the hierarchy of T cell responses change over time after a single immunization? (3) Do homologous or heterologous immunizations affect the T-cell hierarchy? (4) Is the hierarchy influenced by the timing between prime and boost?

## 2 Material and methods

### 2.1 Vectored vaccines

Human serotype 5 Ad and MVA vectors expressing the transgene ME.TRAP have been described previously [13]. The insert ME.TRAP encodes the *Plasmodium falciparum* TRAP and the immunodominant BALB/c H-2Kd epitope pb9 (CS252–260, SYIPSAEKI) from the *P. berghei* circumsporozoite protein.

### 2.2 Mice and immunizations

Animal experiments were performed in accordance with the U.K. Animals (Scientific Procedures) Act, 1986 and associated guidelines and EU Directive 2010/63/EU for animal experiments. Procedures were approved by the University of Oxford Animal Care and Ethical Review Committee. Groups of six 6 week-old female BALB/c mice (Harlan, UK) were injected intradermally in the ear pinna with 10^6^ pfu for MVA or 5 × 10^9^ vp for Ad, under short anesthesia using isoflurane. Ad + MVA vector mixtures (further referred as ‘Mix’) consisted of a single preparation containing both vectors at the same concentrations as each vector alone. All vaccines were injected in a final volume of 50 μl of phosphate-buffered saline.

### 2.3 Ex vivo IFN-γ ELISPOT and peptide targets

Peripheral mononuclear cells (PBMCs) were stimulated with peptides at a final concentration of 1 μg/ml. ELISPOT was performed as previously described [13]. Results were expressed as the average spot forming units (SFU) per million cells. Peptides included the immunodominant Pb9, Ad hexon-specific H-2Kd-restricted epitope hex486–494 KYSPSNVKIA (Kia), Ad DNA-binding protein(dbp)-specific Ld-restricted epitope dbp413–421 LPKLTPFALA (Ala), MVA epitopes previously described E3, F2G [14] and SI9 [15]. Subdominant epitopes were identified in TRAP using 20mer peptides overlapping by 10 and covering TRAP aminoacid (aa) 101–310 from strain T9/96 [16]. This region was previously reported to elicit IFN-γ T-cell responses in BALB/c mice as opposed to peptides covering aa 1–110 and 301–564 (A. Spencer, personal communication and data not shown). Within this region, only epitopes 11 (IRLHSDASKNKEKALIIIRS), 12 (KEKALIIIRSLLSTNLPYGR), 17 (TDGIPDSIKDSLKESRKLSD), 20 (GQGINVAFNRFLVGCHPSDG), 22 (KCNLYADSAWENVKNVIGPF), 25 (TASCGVWDEWSPCSVTCG KG) and 30 (EPLDVPDEPEDDQPRPRGDN) induced detectable responses in at least 1 BALB/c mouse and are represented in the results.

## 3 Results and discussion

### 3.1 Influence of the vector and time on T-cell epitope hierarchy after a single injection

**Fig. 1:**
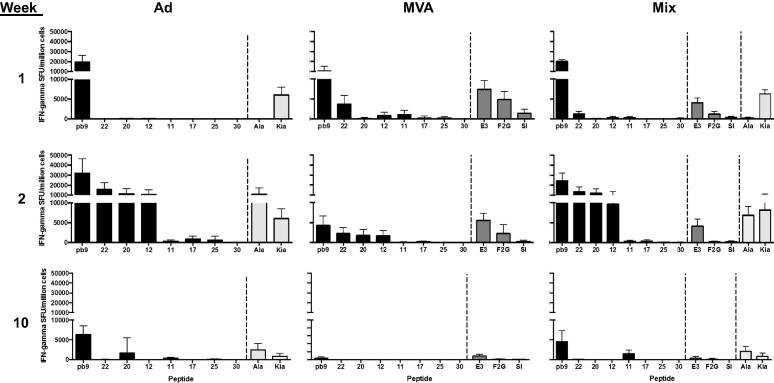
T-cell responses after a single injection. Groups of mice were immunized at day 0 with Ad-METRAP, or MVA-METRAP or a mix of both (Mix), as indicated on the top. IFN-γ responses were measured in individual mice 1, 2 and 10 weeks post a single injection (as indicated on the left) by ELISPOT. Results are presented as the mean and SD for each group and each peptide.

After a single injection with either Ad or MVA, or a mixture of both, T-cell responses were measured in blood at the peak of response, at week 1 for MVA and week 2 post-immunization for Ad [10], and after the contraction phase (week 10) (Fig. 1). Results are presented on a two-segment scale so that both the dominant (10,000–50,000 SFU/million cells) and subdominant (0–10,000 SFU/million cells) responses can be observed for all groups. One week after immunization, Ad induced responses only to the dominant epitope from the transgene, pb9, while responses induced by MVA were lower but broader, targeting peptides 22, 12 and 11 in addition (Fig. 1, top panels). Responses to the vector itself also differed: MVA epitope E3 was as strong as Pb9 in the MVA-immunized group, while the Ad hexon Kia was subdominant in the Ad group. Interestingly, the mixed modality combined the advantages of both vectors: a strong Pb9 response, detectable responses to peptides 22, 11 and 12 and low responses to the MVA vector epitopes. A week later (week 2), responses to the subdominant epitopes in the transgene were detected in the Ad-immunized group (peptides 22 > 20 > 12 > 17 > 25), with higher responses but similar hierarchy as compared with MVA immunization (Fig. 1, middle panels, responses to peptides ranked 22 > 20 > 12 > 17 > 25 in the MVA-immunized group). Of note, the response to the Ad dbp epitope Ala only appeared at week 2 and became dominant over the hexon epitope Kia, reflecting the late appearance of dbp being expressed from the vector while the hexon is present on the vaccine composition [17]. Responses had contracted after 10 weeks from injection. In the MVA-vaccinated animals, viral vector epitope E3 dominated and only the transgene pb9 epitope induced a detectable response (Fig. 1, bottom panels). In contrast, the response remained dominated by Pb9 in the Ad-vaccinated animals, but the subdominant hierarchy was modified, with peptides 20 and 11 still inducing detectable responses, while the response to peptide 22 was lost.

These results suggest that the hierarchy and breadth of T-cell epitope dominance in the transgene is not influenced by the vector, but rather by the timing of analysis after immunization, and the hierarchy changes overtime, which likely reflects affinity maturation and T cell proliferation [18].

### 3.2 Homologous boosts influence the hierarchy of T-cell responses

**Fig. 2:**
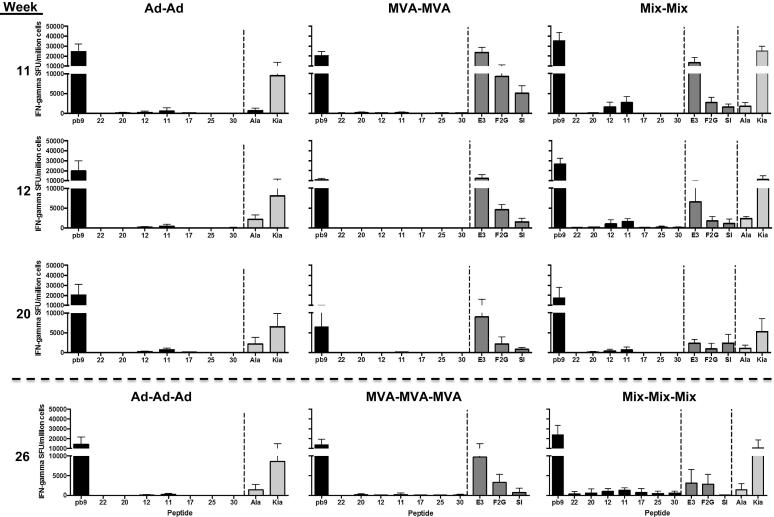
T-cell responses after multiple injections with a 10 weeks interval. Groups of mice were immunized at week 0 with Ad-METRAP, MVA-METRAP or a mix of both (Mix), and boosted at week 10 with the homologous vaccine as indicated on the top. IFN-γ responses were measured in individual mice 1, 2 and 10 weeks post the week 10 boost, as indicated on the left (weeks 11, 12 and 20 post first injection). Mice were boosted at week 24, and the results 2 weeks post this third injection are displayed in the bottom panels (week 26).

To establish whether the T-cell hierarchy is modified after homologous boosting, the same vector was administered after 10 weeks. Responses were measured 1 and 2 weeks post second injection and 10 weeks later (weeks 11, 12 and 20, respectively) (Fig. 2). A reduction in breadth of T-cell responses was observed, focusing only on the dominant epitopes (Fig. 2): pb9 in the transgene, E3 in the MVA-MVA group, and Ad hexon Kia in the Ad-Ad and Mix-Mix groups dominant over dbp at all time points. This reflects the neutralization of the second Ad injection, resulting in lower expression of the transgene and Ad-encoded proteins (as opposed to hexon contained in the preparation) [19] (Fig. 2). The Mix-Mix modality induced a broader response that included peptides 12 in addition to 11, but responses to peptides 22, 20 and 17 were not boosted at all. The responses detected at peak were longer lived than after a single injection, as pb9 and peptide 11-specific responses were detectable at higher levels 10 weeks post boost (week 20, Fig. 2), compared to 10 weeks post prime (Fig. 1).

Therefore the timing difference between 1 week and 2 weeks post injection is not observed anymore after a homologous boost, reflecting the rapid expansion of memory T-cells rather than *de novo* induction [3]. The T-cell hierarchy is modified by repeated homologous immunization, with a clear narrowing and focus on dominant epitopes from the transgene or the vector. Of note, after a third homologous injection performed at week 24, Ad was completely neutralized (no boosting effect was observed as compared with week 20), and only MVA (alone or in the Mix) was able to boost the T cell responses (Fig. 2, bottom panels), suggesting less anti-vector immunity in agreement with previous observations [13].

### 3.3 Effect of prime boost interval and heterologous vaccination on T-cell breadth

**Fig. 3:**
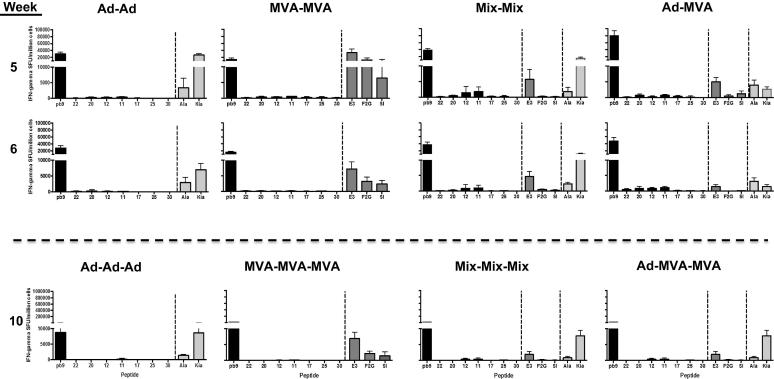
T-cell responses after multiple injections with a 4 weeks interval. Groups of mice were immunized at week 0 with Ad-METRAP, MVA-METRAP or a mix of both (Mix), and boosted at week 4 with the homologous or heterologous vaccine as indicated on the top. Responses were measured in individual mice 1 and 2 weeks post the second injection, as indicated on the left (week 5 and 6). Results are presented as the mean and SD for each group and each peptide. Mice were boosted at week 8, and the results of T-cell ELISPOT 2 weeks post this third injection are displayed in the bottom panels (week 10).

To assess whether narrowing of T-cell responses could be avoided with a shorter vaccination regime, we selected a 4-weeks interval shown to be suitable for eliciting T-cell response to dominant epitopes and suitable for human vaccine deployment. Groups of mice were injected at week 4 with a homologous or heterologous boost (Ad-MVA, Fig. 3). Responses were similar in both regimens (10-weeks and 4-weeks intervals) at 1 or 2 weeks post boost (comparing Fig. 2, top panels with Fig. 3, top panels). The heterologous Ad-prime MVA-boost induced the strongest response to pb9 (reaching a mean of 79,715 SFU/million cells as compared with 37,848 for Mix-Mix). Modest responses to peptides 20, 12, 11 and 22 were detectable in this group. However, when a third injection was administered 4 weeks later, at week 8, the Ad-MVA-MVA regimen also induced a narrowing of the response to pb9, with responses to peptides 11 and 12 barely detectable (measured at week 10, Fig. 3, bottom panels).

Therefore, the timing interval between prime and boost made no difference on the T-cell hierarchy and breadth. The heterologous prime-boost induced the strongest response to the dominant epitope, reflecting the expansion of memory response overcoming the induction of *de novo* response to the new vector. However, this regimen did not support induction of strong responses to subdominant epitopes. These results suggest that clinical selection of viral vectored-based vaccine regimen should be based on the type of response required for protection. If a strong response to a limited number of epitope targets is required, then our results support the heterologous Ad prime – MVA boost as compared with single Ad or Mix immunization. In addition, the heterologous prime-boost was shown to improve the durability, polyfunctionality and phenotype of the T-cell response to the dominant epitope [10], [13]. Results of this study reflect the difficulty to induce broad T-cell responses, important for vaccines aiming at inducing broad and long-lasting T-cell responses to variable pathogens such as HIV [20].

## Conflict of interest statement

AVSH is named as co-inventor on patents related to recombinant viral vectors for malaria and other indications.
